# Combining Next-Generation Sequencing and Microarray Technology into a Transcriptomics Approach for the Non-Model Organism *Chironomus riparius*

**DOI:** 10.1371/journal.pone.0048096

**Published:** 2012-10-25

**Authors:** Marino Marinković, Wim C. de Leeuw, Mark de Jong, Michiel H. S. Kraak, Wim Admiraal, Timo M. Breit, Martijs J. Jonker

**Affiliations:** 1 Microarray Department and Integrative Bioinformatics Unit, Swammerdam Institute for Life Sciences (SILS), University of Amsterdam, Amsterdam, The Netherlands; 2 Department of Aquatic Ecology and Ecotoxicology, Institute for Biodiversity and Ecosystem Dynamics (IBED), University of Amsterdam, Amsterdam, The Netherlands; 3 Netherlands Bioinformatics Centre (NBIC), Nijmegen, The Netherlands; The Roslin Institute, University of Edinburgh, United Kingdom

## Abstract

Whole-transcriptome gene-expression analyses are commonly performed in species that have a sequenced genome and for which microarrays are commercially available. To do such analyses in species with no or limited genome data, i.e. non-model organisms, necessary transcriptomics resources, i.e. an annotated transcriptome and a validated gene-expression microarray, must first be developed. The aim of the present study was to establish an advanced approach for developing transcriptomics resources for non-model organisms by combining next-generation sequencing (NGS) and microarray technology. We applied our approach to the non-biting midge *Chironomus riparius*, an ecologically relevant species that is widely used in sediment ecotoxicity testing. We sampled extensively covering all *C. riparius* developmental stages as well as toxicant exposed larvae and obtained from a normalized cDNA library 1.5 M NGS reads totalling 501 Mbp. Using the NGS data we developed transcriptomics resources in several steps. First, we designed 844 k probes directly on the NGS reads, as well as 76 k probes targeting expressed sequence tags of related species. These probes were tested for their affinity to *C. riparius* DNA and mRNA, by performing two biological experiments with a 1 M probe-selection microarray that contained the entire probe-library. Subsequently, the 1.5 M NGS reads were assembled into 23,709 isotigs and 135,082 singletons, which were associated to ∼55 k, respectively, ∼61 k gene ontology terms and which corresponded together to 22,593 unique protein accessions. An algorithm was developed that took the assembly and the probe affinities to DNA and mRNA into account, what resulted in 59 k highly-reliable probes that targeted uniquely 95% of the isotigs and 18% of the singletons. Concluding, our approach allowed the development of high-quality transcriptomics resources for *C. riparius*, and is applicable to any non-model organism. It is expected, that these resources will advance ecotoxicity testing with *C. riparius* as whole-transcriptome gene-expression analysis are now possible with this species.

## Introduction

Microarray technology has, 17 years after its introduction [1], become a well-established tool for whole-transcriptome gene-expression analyses [2]. Although under pressure due to the on-going developments in next-generation sequencing (NGS) [3], this technology is anticipated to have a viable future for the coming decade given its current low cost, relatively limited data-handling burden, as well as accepted pre-processing and data analyses methods. In contrast to NGS, microarray technology allows comprehensive though cost-effective transcriptome analyses, making it thus possible to test various experimental conditions and to use many replicates, the latter being a necessity to account for biological variability [4]. The option of purchasing completely custom-made microarrays containing up to 4.2 million spots each with a different oligonucleotide (http://www.nimblegen.com/products/cgh/custom/4.2m/index.html) allows for flexible experimentation including the design and the use of huge probe libraries. Over the years, many ground-braking microarray experiments have been performed with respect to unravelling cellular mechanisms, as well as the discovery of predictive/diagnostic biomarkers [5].

Until recently, microarray studies were mainly restricted to several traditional genome-sequenced model species [6]. This meant that in domains that rely on non-model, i.e. non sequenced, organisms, such as ecology and ecotoxicology, microarray technology was only of limited use [6]. The introduction of NGS and in particular medium-long (300–500bp) read pyrosequencing [7] changed this [8], as it became feasible to develop microarrays for any species of interest [9], [10]. In general, the approach to develop transcriptomics resources for non-model organisms is as follows. NGS reads are generated from mRNA and, by lack of reference genome, *de novo* assembled with one or several transcriptome assemblers. Subsequently, microarray probe libraries are designed that target the assembled sequences (contigs/isotigs) and, depending on the microarray format, all or a selection of the un-assembled reads (singletons) [9]–[14]. This approach has several drawbacks, as the microarray design strongly relies on the transcriptome assembly which varies depending on the assembler used [15], [16] and which can result in modified sequences due to the partial assembly of NGS reads [17], the insertion of bases to fill gaps and the merging of NGS reads that do not belong to the same transcript. Moreover, as there is no biological confirmation of the obtained NGS reads in this approach, probes can be developed against sequences that do not target the intended organism, as they are the result of sequencing errors [18] and/or contamination [19], [20]. The eventual microarray design will therefore include many probes that recognize sequences not present in the target organism. Finally, as this approach relies solely on *in-silico* methods, a fraction of the probes may also perform badly in actual microarray experiments.

**Figure 1:**
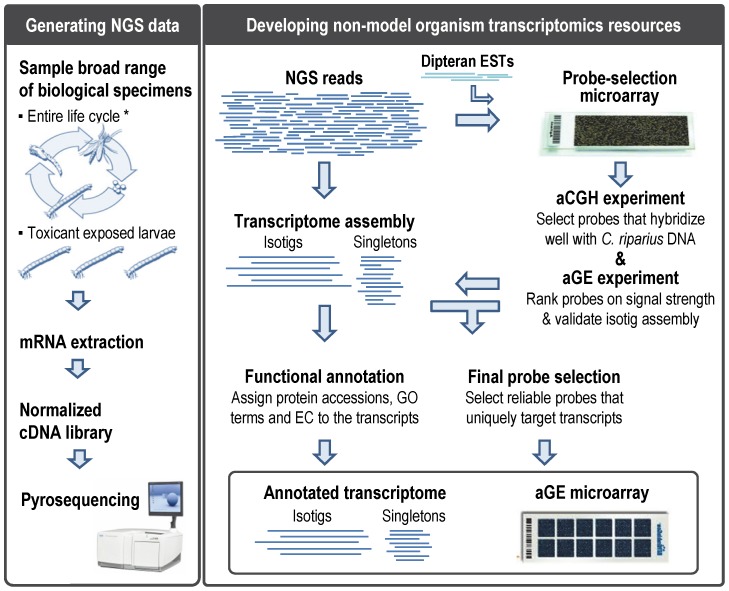
Strategy to obtain non-model organism transcriptomics resources. NGS: Next-generation sequencing; ESTs: Expressed Sequence Tags; aCGH: array-based Comparative Genomic Hybridization; GE: Gene Expression; GO: Gene Ontology; EC: Enzyme Commission numbers. * adapted from http://extension.missouri.edu/explorepdf/agguides/pests/g07402.pdf.

Given the above discussed drawbacks, the aim of the present study was to establish an advanced approach for developing transcriptomics resources for non-model organisms, consisting of an annotated transcriptome and a high-quality microarray. To achieve this, we formulated the following strategy (Figure 1):

- Generate NGS data: Perform a NGS experiment on a normalized cDNA library obtained from a broad range of biological samples of the non-model organism.
- Design probe library: Design up to one million probes targeting all original NGS reads, as well as, expressed sequence tags (ESTs) of related species.
- Assemble the NGS reads. Assemble the NGS reads into a transcriptome for downstream probe selection and functional annotation.
- Select probes with targets in the genome: Conduct an array-based comparative genomic hybridization (aCGH) experiment with a probe-selection microarray that contains all the designed probes, to select probes that hybridize well with the genomic DNA (gDNA) of the non-model organism.
- Select probes for standard mRNA analysis: Conduct an array-based gene-expression (aGE) experiment with the probe-selection microarray to further select probes according to their 3′ location in mRNA, their signal-intensity and their ability to uniquely interrogate the assembled transcripts. The final probe library targets all non-model organism transcripts that can be uniquely targeted while keeping the number of probes to a minimum.
- Finalize transcriptomics resources: Functionally annotate all transcripts using the annotation tool Blast2GO® [21] and determine which transcripts are targeted by the high-quality GE microarray.

As microarrays are gaining importance in ecotoxicology [22], [23], we applied this approach to a non-model organism commonly used in ecotoxicology for which till date no microarray has been developed and for which very limited sequence data can be found in public repositories, even though the transcriptome of this non-model species has recently been published [24]. The non-biting midge *Chironomus riparius* (Insecta: Diptera) is a member of the Chironomidae family, which are the most widely distributed and often most abundant insects in freshwater ecosystems [25]. Consequently, chironomids are routinely used to evaluate and monitor the biological quality of rivers and lakes [26], [27]. Their larvae settle in the sediment, where they remain until they emerge as adults. The short life-cycle and ease of rearing have also made *C. riparius* a commonly used species in sediment ecotoxicology [28], [29] with currently four standardized OECD guidelines being available for acute and chronic toxicity tests [30]–[33]. Assessing effects on life cycle endpoints has proven to be an effective method for deriving effect concentrations for environmental risk assessment [34]. However, life cycle effects are not supported by a mechanistic insight in the toxicants mode of action nor the physiological changes that occur in toxicant exposed midges. Studies that measure effects at lower levels of biological organization, such as the transcriptome, are therefore required [35], [36].

## Results and Discussion

### Generation of Transcriptome Next-generation Sequencing Data

Non-model organisms have no or limited genomics data. Due to the size and complexity of the genome, sequencing the transcriptome is a practical alternative to obtain genomics data for such species [8]. The obvious drawback is that only sequences from genes that are expressed in the sequenced samples will become available. Therefore broad sampling, as well as normalization of the pooled sample, i.e. reducing the frequency of highly abundant transcripts, is highly recommended (Figure 1). For *C. riparius* we included all life cycle stages, i.e. egg ropes, all four larval stages, pupae and male and female adults. Considering *C. riparius* use in sediment ecotoxicology [28], [29], we also included larvae exposed to different concentrations of several model toxicants (Table 1, extended version in Table S1). These specimens were used to synthesize a normalised cDNA library that yielded 1,549,146 NGS reads with a total length of 500,673,325 bp which is in line with the specification of the 454 NGS platform and which is more than ten times the amount of reads and base pairs previously reported by Nair et al. [24] who obtained, respectively, 138,091 NGS reads and 49,774,676 bp by pyrosequencing toxicant exposed 4^th^ instar larvae.

**Table 1:** *C. riparius* sample list summary.

Specimen	Pre-exposed	Exposed	Time/Dose range	Number
Egg ropes	n.a.	n.a.	<1 h–72 h post laying	16
Larvae (Instar I-IV)	n.a.	n.a.	<1 day–14 days post hatching	16
Pupae	n.a.	n.a.	14–16 days post hatching	4
Adult males	n.a.	n.a.	<1 h–60 h post emerging	16
Adults females	n.a.	n.a.	<1 h–60 h post emerging	16
Larvae	n.a.	Cadmium	0.5–4.0 mg Cd/kg dw	4
Larvae	n.a.	Copper	10–40 mg Cu/k/dw	4
Larvae	n.a.	Tributyltin	0.5–3.0 mg Sn/kg dw	4
Larvae	n.a.	Phenanthrene	50–400 mg Phe/kg dw	4
Larvae	Cadmium	Cadmium	0.5–4.0 mg Cd/kg dw	4
Larvae	Copper	Copper	10–40 mg Cu/kg dw	4
Larvae	Tributyltin	Tributyltin	0.5–3.0 mg Sn/kg dw	4
Larvae	Phenanthrene	Phenanthrene	50–400 mg Phe/kg dw	4

* n.a.: not applicable.

### Generation of Microarray Probe Library

In our microarray approach, we started by designing a huge probe-library directly on all adapter trimmed NGS reads that were longer than 60 bp and that did not contain unknown bases in their sequence, using the in-house developed NGS array designer (http://mad1.science.uva.nl/projects/NGSdesigner). Designing probes against NGS reads, instead of the assembled contigs/isotigs ensured that the designed probes did not target sequences that might have been modified during the assembly process. The probe-library was subsequently extended with probes designed against publically available ESTs of closely related species. These probes could be an enrichment as they might target conserved sequences that were not present in the sequenced sample. For *C. riparius*, this resulted, after testing for cross-hybridization, in 919,821 probes. 843,837 NGS read designed probes targeted almost al NGS reads, while 75,984 probes were designed against ESTs belonging to the genus Chironomus and the closely related dipteran species *Anopheles gambiae*, *Anopheles darlingi*, *Anopheles funestus*, *Aedes aegypti* and *Culex quinquefasciatus*.

### Assembly of Transcriptome NGS Data

For the downstream probe selection procedure, as well as the annotation of the non-model species transcriptome, the NGS reads had to be assembled. For *C. riparius* we used Newbler (v2.5.3.) which is a de-facto standard assembler for NGS reads generated by pyrosequencing [37] and which has been shown to perform best assembling such NGS reads *de novo* into a transcriptome [15]. An overview of sequencing and assembly statistics is given in Table 2. From the ∼1,5 million trimmed NGS reads, 87.2% was fully or partially assembled, 8.8% could not be assembled and was labelled singleton, while the remaining 4.1% was discarded as the NGS reads did not meet the required quality standards. While the large number of singletons undoubtedly contained fragments of rare transcripts [38], [39], we suspected that a substantial portion was the result of NGS errors, artefacts of cDNA library preparation and/or contaminants from other sources. Especially the latter option seems plausible, since entire larvae including their gut flora were used for the cDNA library preparation. To keep track of possible differences between the two transcript sets, we kept the isotigs and singletons separated during our entire study.

**Table 2:** *C. riparius* transcriptome sequencing and assembly statistics.

Category	Sequences	Base pairs
*Sequencing statistics*		
Raw NGS reads	1,549,146	500,673,325
NGS reads 1	1,540,849	459,548,838
NGS reads N50 2	–	347
*Assembly statistics*		
Assembled NGS reads	1,342,920	409,774,142
Discarded NGS reads 3	63,401	10,253,972
Singletons	135,082	39,520,724
Singleton N50 2	–	343
Contigs ("exons")	27,334	21,898,252
Contig N50 2	–	1,161
Mean # NGS reads/contig	59.0	–
Isotigs ("transcripts")	23,683	32,429,684
Isotig N50 2	–	1,886
Mean # contigs/isotig	1.9	–
Isogroups ("genes")	18,514	–
Mean # isotigs/isogroup	1.3	–

1 after trimming of adaptor sequences;

2 N50 is a weighted median, such that half the bases are contained in sequences equal to or larger than the N50 length;

3 NGS reads that were discarded during the assembly process because they were too short (<50 bp), contained repeats or were marked as outliers by the Newbler assembler.

Since the Newbler assembler takes alternative splicing into account, the *C. riparius* transcriptome assembly consisted of ∼27 k contigs (“exons”) that were incorporated into ∼23 k isotigs (“transcripts”, average length 1,369 bp), which in turn were grouped into ∼18 k isogroups (“genes”). 66.8% of the isotigs consisted of a single contig, while the others contained up to 13 contigs. Isotigs that shared contigs were grouped together into isogroups, which resulted in 85.8% with one, 10.5% with two, and 3.7% with three or more isotigs. Correcting for 26 contigs that were not translated by the Newbler assembler into isotigs, we defined the *C. riparius* transcriptome as the total of 23,709 isotigs and 135,082 singletons.

### aCGH Experiment to Select Relevant Microarray Probes using gDNA

To select the most reliable and biological relevant probes from the previously designed probe-library, a probe-selection microarray was developed that contained the probe-library and ample negative-control probes that did not recognize any sequence in GenBank. Performing an aCGH experiment allowed subsequently the identification of probes that hybridized with the gDNA of the non-model organism. Assuming a limited chance on random homology, the aCGH experiment was expected to considerably clean-up the NGS data, eliminating NGS reads that originated from contamination or technological errors, while simultaneously selecting relevant probes for the final non-model species GE microarray. It is recognized, that his procedure would also select against probes targeting exon spanning sequences, however, due to the large number of probes this was not considered a problem.

For *C. riparius*, we developed a 1 M probe-selection microarray that contained the 919,821 probes from the probe-library and 40.000 negative control probes. This probe-selection microarray was used in an aCGH experiment to analyse the gDNA of *C. riparius* as well as the gDNA of *A. gambiae* that served as a positive control. It turned out that there was a correlation between the GC-content of the negative control probes and their signal-intensity, i.e. above a GC-content of 45% a steady increase in signal intensity was observed (Figure S1). This prompted us to limit the CG-content for all probes to a maximum of 50%, what resulted in 816,270 *C. riparius* NGS-read probes, 42,636 dipteran-specific probes, and 19.000 negative-control probes.

**Figure 2:**
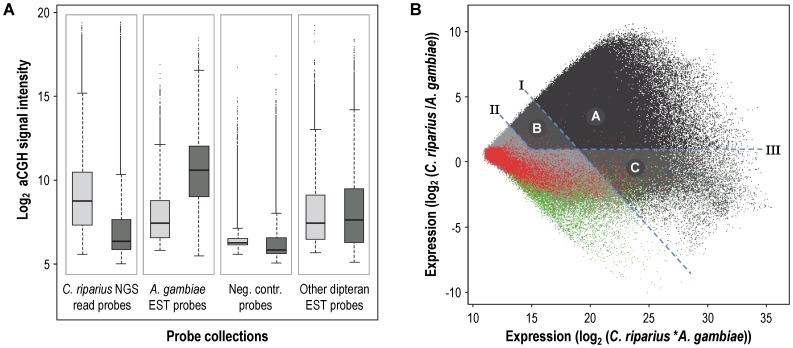
Array-based comparative genomic hybridization (aCGH) experiment. (A) Box- and-whisker plot summarizing the obtained log_2_ signal intensity distributions for the four indicated probes collections, with the light grey boxes representing the *C. riparius* aCGH signal and the dark grey the aCGH *A. gambiae* signal. (B) MA-plot of the aCGH data. The dots with the different shades of grey represent the entire probe-library (with a GC-content below 50%). The three defined signal-intensity parameters are indicated by the dashed blue line and the captions I, II, III. The three categories containing the selected probes are indicated by different shades of grey and the letters A, B and C. The red dots are the negative control probes and the green dots the positive control (*A. gambiae* EST) probes.

As expected, the NGS-read probes showed stronger signal intensities after hybridisation with *C. riparius* than *A. gambiae* gDNA (Figure 2A). The opposite was true for the probes designed against *A. gambiae* ESTs. In fact, the *A. gambiae* aCGH signal-intensities of the NGS-read probes were in the range of that of the negative controls, indicating a substantial genomic difference between the malaria mosquito *A. gambiae* and the non-biting midge *C. riparius*. To select probes that hybridized well to the *C. riparius* gDNA, we compared for all probes the log_2_-ratio (*C. riparius/A. gambiae*) of the aGCH signal intensities with the summed log_2_ (C. riparius*A. gambiae) signal intensities in a MA-plot (Figure 2B). The negative- and positive (*A. gambiae*) control probes behaved as expected, both with low *C. riparius* aGCH signal intensities and only for the positive control probes high signal intensities for the *A. gambiae* gDNA. The distributions of the probes designed against the other dipteran species, with the exception of the Chironomus spp, corresponded to the pattern observed for the *A. gambiae* designed probes (Figure S2).

Based on the distributions of the control probes (Figure 2B), we defined signal-intensity parameters that allowed a conservative selection of *C. riparius* gDNA specific probes: parameter I was a *C. riparius* log_2_ signal of 10, separating probes with a strong signal when hybridized with *C. riparius* gDNA; parameter II was a *C. riparius* log2 signal of 8, separating probes with an intermediate signal when hybridized with *C. riparius* gDNA; and parameter III was a log_2_ *C. riparius*/*A. gambiae* signal ratio of 1, separating probes with a higher signal to *C. riparius* than to *A. gambiae* gDNA. Using these parameters we defined categories to select probes: Category A contained probes above parameter I and III, which are probes that gave a strong and specific *C. riparius* gDNA signal (217,878); Category B contained probes between parameter I and II and above III, which are probes that gave an intermediate and specific *C. riparius* gDNA signal (206,608); and Category C contained probes above I and below III, which are probes that gave a strong and non-specific *C. riparius* gDNA signal (42,379). These categories contained a total of 466,865 reliable probes. The validity of the selection parameters was confirmed by applying the same signal-intensity parameters to the negative and positive control probes, what resulted in the selection of only 2.7% of the negative control probes (Figure 2B) and 86.6% of the positive control probes, with 2,860, 1,428 and 165 positive control probes in the categories A, B, and C, respectively.

Hence, with this approach we were able to reduce our initial pool of probes with 49.6%. However, despite this reduction in probes, still 98.2% isotigs and 60.2% singletons were targeted. This effectively means that the aCGH experiment selected against bad-performing probes, rather than bad NGS reads. A bad-performing probe can be caused by low affinity to the target due to small NGS errors, exon spanning target sequences, reads belonging to other species and sequence-specific microarray technology anomalies.

### aGE Experiment to Further Select 3′ Located Probes using mRNA

**Figure 3:**
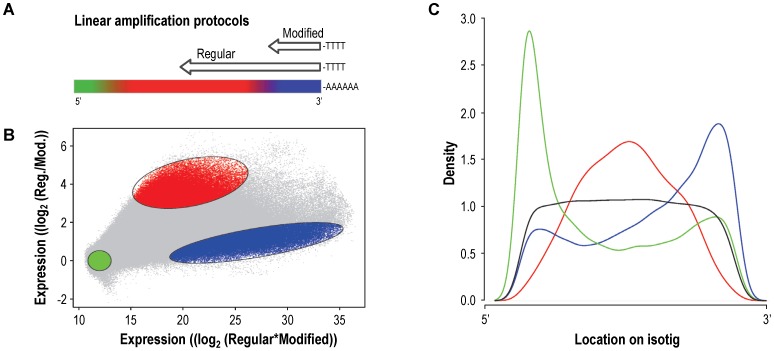
Array-based gene expression (aGE) experiment. (A) Schematic representation of the two mRNA linear amplification protocols. The coloured bar represents the mRNA with the 3′ polyA tail indicated by the stretch of A’s. The arrows represent the amplified cDNA products obtained for the regular procedure and the modified procedure, with the length of the arrows indicating the length of the synthesized cDNA’s. (B) MA-plot of the aGE data. The light grey dots represent all aCGH selected probes. The three coloured regions are expected to contain probes targeting transcripts at the 3′ side (blue), probes targeting the middle of the transcripts (red) and probes targeting the 5′side as well as probes with no target transcripts (green). (C) Density plot where the relative position of the three probe populations on the isotigs is demonstrated. The colours of the lines correspond to the colours used in panels A and B. The black line represents a random selection of probes that covers, as expected, the isotigs evenly over the entire length.

After the DNA check by the aGCH experiment, we used mRNA derived from the non-model organism in the probe-selection procedure. Given the relative small average size of the isotigs and the many singletons, it was fair to assume that many transcripts in our example represented incomplete mRNA sequences, something that will often be the case in *de novo* assembled non-model organisms transcriptomes. As the standard aGE protocol uses linearly amplified cDNA, i.e. the mRNA is amplified using oligo-dT primers from the 3′-side, only 3′ located probes will be useful for the final aGE microarray. Identifying probes against the most 3′ located target sequences in the mRNA was possible by combining a standard linear amplification protocol that yielded 3′-end biased labelled material, with a modified linear amplification protocol that yielded highly 3′ restricted material due to the incorporation of dideoxynucleotides during the cDNA synthesizing step (Figure 3A). For this experiment, the same probe-selection microarray was used as in the aCGH experiment.

For *C. riparius*, two aGE samples were synthesized from the same mRNA pool that was pyrosequenced, using the regular and modified linear amplification protocols (Figure 3A). Comparing the two samples on the 1 M probe-selection microarray resulted in three populations of probes. First, probes that showed a good intensity signal in both procedures: these probes recognized targets located 3′ in mRNA that was expressed at a high enough level to be detected by microarray technology. Second, probes that performed well in the regular protocol, but not in the 3′ restricted protocol: these probes recognized targets located more 5′ in mRNA that was expressed at a sufficient level. Third, probes that performed badly in both protocols: these probes recognized targets either located too much 5′ in mRNA to be amplified by either protocol, targets from genes that were expressed below the detection level of microarray technology, or targets from genes that were not present in the tested transcriptome, i.e. NGS contamination or erroneous sequences. This approach worked quite well and revealed that the anticipated three probe populations could be identified (Figure 3B). To demonstrate that these populations indeed represented 5′, middle and 3′ located mRNA target sequences, we determined the relative position of their targets on the assembled isotigs (Figure 3C). Compared to a set of randomly chosen probes, the distributions showed a strong preference for the expected location. Hence, there were likely probes targeting 5′, middle and 3′ located sequences for each mRNA in this NGS data set. Since the different probe populations could not clearly be separated, the aGE data was used to rank the probes based on their signal strengths and thus the target location on the NGS-defined transcripts.

To achieve our goal and design a cost-effective 3′-primed gene-expression microarray, we aimed to keep the number of reliable and biologically relevant probes to a minimum, while making sure that as many as possible transcripts were uniquely targeted. For this, a selection algorithm was defined that was executed for both isotig and singleton sets. This algorithm started by categorizing the probes for isotigs using blastn: probes were defined having good matches to isotigs, if the bit score was >80. Probes with no good match to any isotig were discarded. Then, probes were selected only if they were unique to one isogroup. Before the final selection, probes were evaluated to contain only stretches of 7 identical nucleotides or less, as well as 5 subsequent di-nucleotides or less. The selected probes were ranked according to their aGE signal intensity. Starting with the highest-ranked probe, probes were selected for each isogroup until all isotigs within each isogroup were targeted by at least one probe. The same algorithm was then applied to the singleton set.

Applying this algorithm to our *C. riparius* aGE data, we obtained a final probe library that consisted of 59,409 validated probes uniquely targeting 22,507 isotigs, (corresponding to 17,403 isogroups) and 23,782 singletons.

### Finalization of the Transcriptomics Resources

To allow down-stream analyses and interpretation of gene-expression studies, functional annotation of the transcriptome was needed. Functional annotation of the isotigs and singletons was performed independently, starting with a blastx search of both sets against the GenBank non-redundant (nr) protein database followed by a Blast2GO® analyses identifying relevant Gene Ontology (GO) terms [40] and unique enzyme commission (EC) numbers.

For *C. riparius*, we used a lenient blastx e-value threshold of 1*e^−3^ and found matches to homologous proteins for 71.0% of the isotigs and 17.9% of the singletons (Table S2). Screening those blastx hits for identical protein accessions, showed that 50.0% of all isotigs and 8.9% of the singletons corresponded to unique protein accessions numbers. From the blastx results we identified a total of 22,593 unique protein accessions of which only 6% were found in both transcript sets. The fact that the isotigs and singletons almost equally contributed to this total set of unique protein accessions indicated that the assembly was exhaustive and thus successful, as well as that biologically relevant information was indeed present in the singletons. The number of unique protein accessions obtained in the present study is higher than the 9,512 unique protein accessions previously reported by Nair et al. [24], most probably because they pyrosequenced less deep and because their biological sample was less diverse as it merely consisted of 4^th^ instar *C. riparius* larvae. Since the genome sequence of *C. riparius* is not elucidated we cannot determine the exact coverage of the transcriptome. However, considering that the sequenced genomes of the closely related mosquitos *A. aegypti, C. quinquefasciatus* and *A. gambiae* are predicted to have 16,789, 18,883, respectively, 13,133 transcripts [41], it seems likely that we covered a substantial part of the *C. riparius* transcriptome.

**Figure 4:**
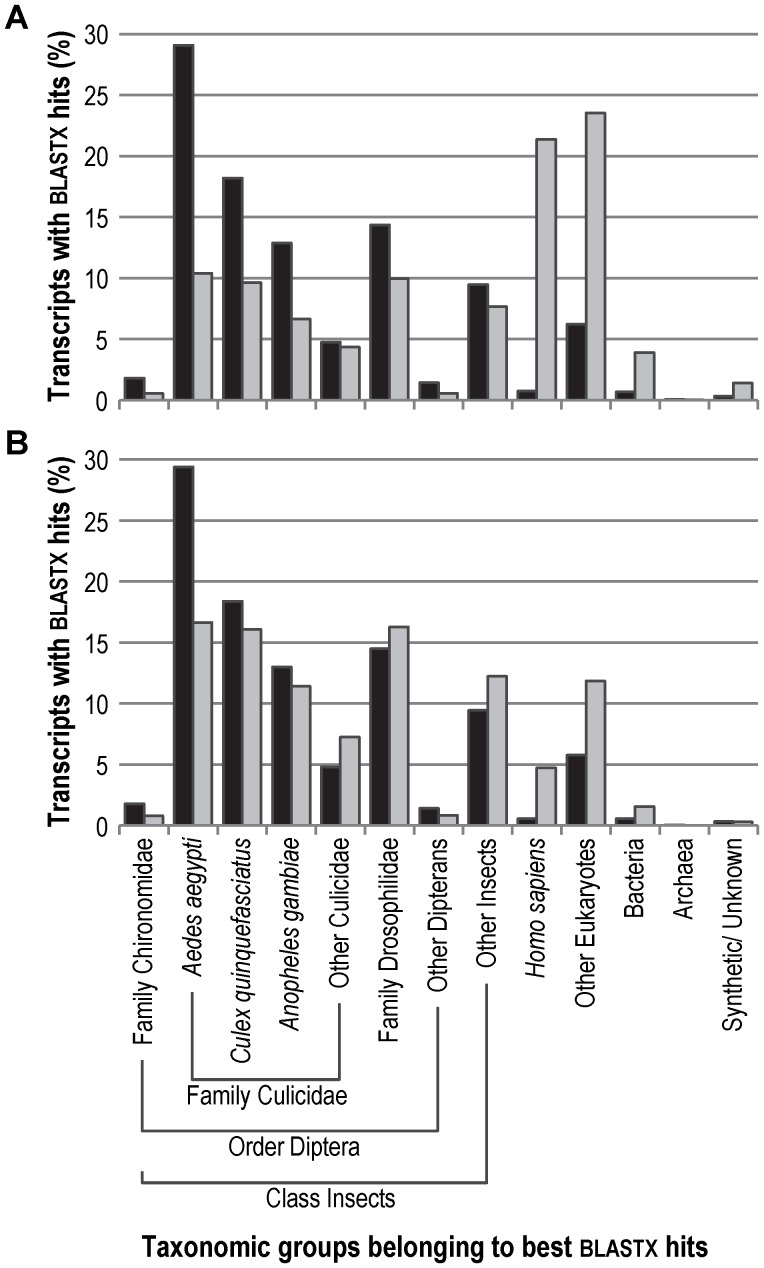
Taxonomic distribution of the best blastx hits matching *C. riparius* transcripts. Distribution of the best blastx hits that were matched to the isotigs (black) and the singletons (light grey) according to their taxonomic origin. (A) All transcripts (isotigs n = 16,824; singletons n = 24,129) that were matched to a BLASTX hit. (B) Transcripts (isotigs n = 16,537; singletons n = 4,7539) that were matched to a BLASTX hit and that are targeted by the final aGE microarray.

Distributing all the best blastx hits over taxonomic groups (Figure 4A), showed, as expected, that the far majority (92.0%) of isotig-matched proteins belonged to known insect proteins, and especially to the dipterans *A. aegypti* (29.1%), *C. quinquefasciatus* (18.2%) and *A. gambiae* (12.9%). This is in concordance with the distribution reported by Nair et al. [24]. For the singleton-matched proteins the distribution was different in that 49.8% related to insect proteins and 45.0% to other eukaryotic species. Only small fractions of the isotigs and singletons matched to prokaryote proteins (0.7% and 3,9%), which indicates that the chironomid gut flora did not substantially contaminate the NGS data. Since 0.7% of the isotigs and 21.4% of the singletons matched human proteins, we performed a blastn (nucleotide) search against the human genome and transcriptome data available at NCBI, to further estimate the potential human contamination of the NGS data. We detected that 3.5% of NGS reads showed a strong similarity to human sequences over the entire read length and that 94.0% of these ‘human’ NGS reads remained unassembled, i.e. became singletons. As these sequences could also represent conserved genomic sequences, we chose not to remove them from our NGS data.

**Figure 5:**
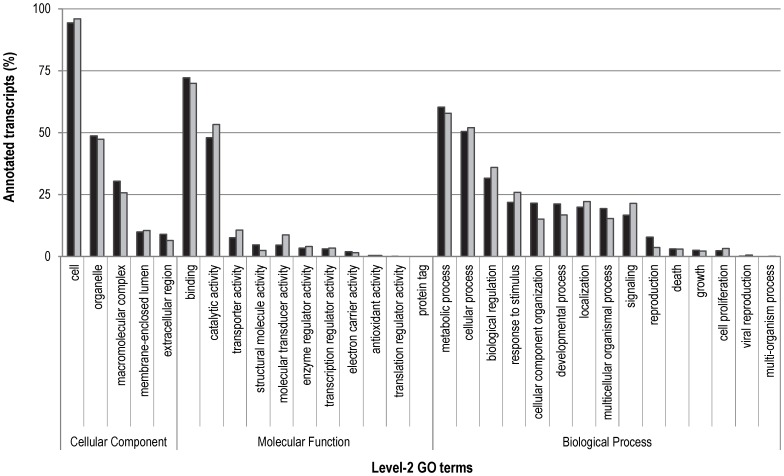
Gene Ontology (GO) terms obtained for *C. riparius* transcripts. The data represents the distribution of the annotated isotigs (black) and the annotated singletons (light grey) over the various level-2 GO terms. Each bar represent the number of annotated transcripts associated with the specified level-2 GO term as a percentage of the total number of annotated transcripts belonging to the higher-ranked GO category, i.e. cellular component (isotigs n = 8,380; singletons n = 9,277), molecular function (isotigs n = 10,663; singletons n = 11,359) and biological process (isotigs n = 6,249; singletons n = 7,343).

Assigning functional categories to the *C. riparius* transcriptome, we were able to annotate 11,895 (50.2%) isotigs and 12,662 (9.4%) singletons with ∼55 k and ∼61 k GO terms, respectively (Table 3 and Table S3). The distribution over the various GO terms was remarkably similar for the isotigs and singletons (Figure 5) and may suggest that the observed patterns at least partially depended on the abundance of certain GO-terms. To visualize the interaction of the annotated transcripts, we assigned with Blast2GO®, 688 unique EC numbers to 2,973 isotigs and 3,611 singletons (Table 3 and Table S3) and found 126 pathways in the Kyoto Encyclopedia of Genes and Genomes (KEGG) database [42].

**Table 3:** *C. riparius* transcriptome annotation summery.

Category	Isotigs	Singletons
Total number of transcripts	23,709	135,082
Transcripts with blastx match	16,824	24,129
Transcripts assigned GO terms	14,290	17,698
Annotated transcripts	11,895	12,662
▪ with GO terms for biological	6,249	7,343
processes *(# GO terms)*	*(25,689)*	*(23,976)*
▪ with GO terms for molecular	10,663	11,359
functions *(# GO terms)*	*(19,443)*	*(23,304)*
▪ with GO terms for cellular	8,380	9,277
components *(# GO terms)*	*(10,172)*	*(13,540)*
Transcripts with Enzyme Codes	2,973	3,611

The final GE microarray (Gene-Expression Omnibus accession numbers GPL15611) targeted 22,507 isotigs and 23,782 singletons. Of these targeted transcripts, 73.5% isotigs and 20.0% singletons had a blastx hit, of which 71.4% and 88.3% matched to a unique protein accessions. Importantly, our selected probes targeted 94.9% of the isotigs and 17.6% of the singletons, which covered 98.6% and 34.9% of the unique protein accessions, respectively. The taxonomic distribution of the targeted isotigs was almost the same of that of the entire set of isotigs with 92.7% matching insect proteins. However, for the singletons the percentage that matched insect proteins increased substantially from 49.8% to 82.2% (Figure 4B). As aimed for, the percentage of ‘human contamination’ was substantially reduced in both transcript sets.

Thus by combining NGS and microarray technology we succeeded in designing an annotated high-quality microarray suited for whole-transcriptome gene-expression analysis in a non-model organism. For *C. riparius*, we selected from a 925 k probe library 59 k highly-reliable probes that have been proven to perform well in both aCGH and aGE experiments. While we designed probes directly on the NGS reads, our approach is also suitable for validating probe libraries designed against assembled transcripts. Concluding, we now have valuable *C. riparius* transcriptomics resources, i.e. an annotated transcriptome and a 135 K 3′-primed gene-expression microarray, that can advance ecotoxicity testing with *C. riparius* as whole-transcriptome gene-expression analysis are now possible with this species.

## Materials and Methods

### Test Organism, Culturing Conditions and Sample Selection

The *Chironomus riparius* specimens originated from the University of Amsterdam’s in-house laboratory culture and were maintained on artificial sediment at 20±1°C, 65% humidity and a 16∶8 h light: dark photoperiod [28], [29]. The genetic fidelity of this *C. riparius* laboratory culture was previously confirmed by mitochondrial cytochrome oxidase I (COI) gene sequencing [43] The sample list, including all life cycle stages and toxicant-exposed larvae, is provided in Table S1. Each sample was immediately snap frozen in liquid nitrogen and stored at –80°C until processing.

### RNA Isolation, cDNA Library Construction and Next-generation Sequencing

The frozen samples were pooled and homogenization in liquid nitrogen. Total RNA was extracted using the RNeasy Mini kit (Qiagen) with an on-column DNase (Qiagen) digestion to remove traces of genomic DNA. RNA integrity was examined using a RNA 6000 Nano chip on a 2100 Bioanalyzer (Agilent Technologies), while the RNA yield was determined on a NanoDrop ND-1000 UV-VIS spectrophotometer (Thermo Fisher Scientific). An aliquot of the total RNA sample was send to GATC Biotech (Konstanz, Germany) where a normalized cDNA library was prepared and sequenced using Titanium chemistry on a GS FLX Instrument (Roche Diagnostics) according to manufacturer’s protocol. Detailed information is presented in Appendix S1.

### *De novo* Transcriptome Assembly and Functional Annotation

NGS reads were, after removal of adapter sequences, assembled with Newbler v2.5.3. (Roche) in the *de novo* mode using default assembly parameters. The obtained isogroups and isotigs, as well as, the remaining singletons were renamed in the format of “CripIG000001”, “CripIT000001”, “CripSI000001” with “Crip” standing for *C. riparius*, “IG” for isogroup, “IT” for isotig, “SI” for singleton, and “000001” for an arbitrary assigned number. The *C. riparius* transcripts, i.e. isotigs and singletons, were functionally annotated using the Blast2GO® suite [21], [44]. Detailed information of the functional annotation procedure is presented in Appendix S1.

### Array-based Comparative Genomic Hybridization (aCGH)

The designed 1×1 M probe-selection microarray was obtained from Agilent Technologies and hybridized with *C. riparius* and *A. gambiae* genomic DNA (gDNA). gDNA was extracted from 30 pooled fertilized *C. riparius* egg ropes, respectively, 30 pooled unfed *A. gambiae* adults. To keep contamination of the *C. riparius* gDNA sample to an absolute minimum the midges were allowed to deposit the egg ropes in petri dishes filled with clean water. Egg ropes not older than 30 minutes were collected, stringently rinsed with clean water and immediately flash frozen in liquid nitrogen. After pooling, gDNA was extracted using a CTAB DNA extraction method that included a RNAse A (Sigma-Aldrich) digestion step for removal of residual RNA [45]. gDNA quality and quantity were determined with gel electrophoresis (0.5% agarose in TAE buffer) and NanoDrop ND-1000 measurements. 200 ng DNA was amplified and labelled by strand displacement amplification. Concentrations of amplified products were measured on the NanoDrop ND-1000 and qualified on the BioAnalyzer with the DNA 1000 Kit (Agilent Technologies). Yield and CyDye incorporation of the final labelled products were measured with the NanoDrop ND-1000 in the Microarray Measurement Mode. The 1 M probe-selection microarray was subsequently hybridized with 10 µg of Cy3 labelled *C. riparius* gDNA and 10 µg Cy5 labelled *A. gambiae* gDNA according to the Oligonucleotide Array-Based CGH for Genomic DNA Analysis manual (Agilent Technologies version 6.3. The microarray was scanned in an ozone-free room on an Agilent G2505CA scanner at 3 µm resolution and the data was extracted with Feature Extraction version 10.7.3.1 (Protocol CGH_107_Sep09). The log_2_ transformed median signals were analysed in R (www.r-project.org). Detailed information is presented in Appendix S1.

### Array-based Gene-expression (aGE)

The pooled *C. riparius* RNA sample, of which an aliquot was pyrosequenced, was used to identify probes corresponding to the 3′-end of *C. riparius* transcripts. 200 ng RNA was taken as input for both a regular, as well as, a modified linear RNA amplification. The regular RNA amplification was conducted with the Agilent Low RNA Input Linear Amplification Kit (Agilent Technologies) according to manufacturer’s recommendations. The modified reaction was conducted using the same kit, however, the first step where the mRNA is primed with an oligo (d)T-T7 primer and converted into double-stranded cDNA with the M-MLV reverse transcriptase, was modified by the addition of dideoxynucleotides (ddNTP) in the deoxynucleotide mix. This modification was expected to yield highly 3′-biased cDNA as incorporation of a ddNTP would prematurely terminate cDNA elongation. The modified amplification procedure is described in detail in the Appendix S1. Amplified RNA was checked for quality and quantity using Bioanalyzer and NanoDrop measurements. Yield and CyDye incorporation of the final labelled products were measured with the NanoDrop ND-1000 in the Microarray Measurement Mode. The 1 M probe-selection microarray was subsequently hybridized with 2.5 µg Cy3 labelled regularly amplified RNA and 2.5 µg Cy5 labelled alternatively amplified RNA according to the Two-Colour Microarray-Based Gene-Expression Analysis manual (Agilent Technologies version 6.5). The microarray was scanned in an ozone-free room on an Agilent G2505CA scanner at 3 µm resolution. The data was extracted with Feature Extraction version 10.7.3.1 (Protocol GE2_107_Sep09). The log_2_ transformed median signals were analysed in R.

### Data Deposition

The *C. riparius* NGS sequence reads were submitted to NCBI Sequence Read Archive (www.ncbi.nlm.nih.gov/sra) under accession number SRX147945. The assembled *C. riparius* isotigs have been submitted to NCBI Transcriptome Shotgun Assembly Sequence Database (www.ncbi.nlm.nih.gov/genbank/tsa) and can be accessed through the GenBank accession numbers KA174710-KA198345. Complete raw microarray data and their MIAME compliant metadata have been deposited at NCBI Gene-Expression Omnibus (www.ncbi.nlm.nih.gov/geo) under accession numbers GPL15610 (1 M microarray) and GPL15611 (135 K microarray).

## Supporting Information

Figure S1 **aCGH signal intensities of the control probes according to GC-content.** Box- and-whisker plot showing the log_2_ *A. gambiae* signal intensity distributions of the positive control probes (light grey) and the negative control probes (dark grey) for 20 GC-bins, each bin corresponding to a GC-content increase of 5%.
(TIFF)

Figure S2 **MA-plots of the aCGH experiment obtained for the various dipteran probe collections.** The grey dots represent the entire probe library, except the negative control probes. The black are the probes targeting the ESTs of respectively (A) Chironomus spp., (B) *Anopeheles darlingi*, (C) *Anopheles funestus*, (D), *Aedes aegypti* and (E) *Culex quinquefasciatus*.
(TIFF)

Table S1 **Detailed** ***C. riparius*** **sample list.**
(DOCX)

Table S2 **blastx results using different e-value cut-offs.**
(DOCX)

Table S3 **The** ***C. riparius*** **transcriptome annotation.**
(7Z) Click here for additional data file.

Appendix S1 **Additional information to the Materials and Methods.**
(DOCX)
