# Supplementary figures and images for: Combining Next-Generation Sequencing and Microarray Technology into a Transcriptomics Approach for the Non-Model Organism Chironomus riparius
Source: PLoS One. 2012 Oct 25;7(10):e48096. doi: 10.1371/journal.pone.0048096 (PMC3485019; doi:10.1371/journal.pone.0048096)

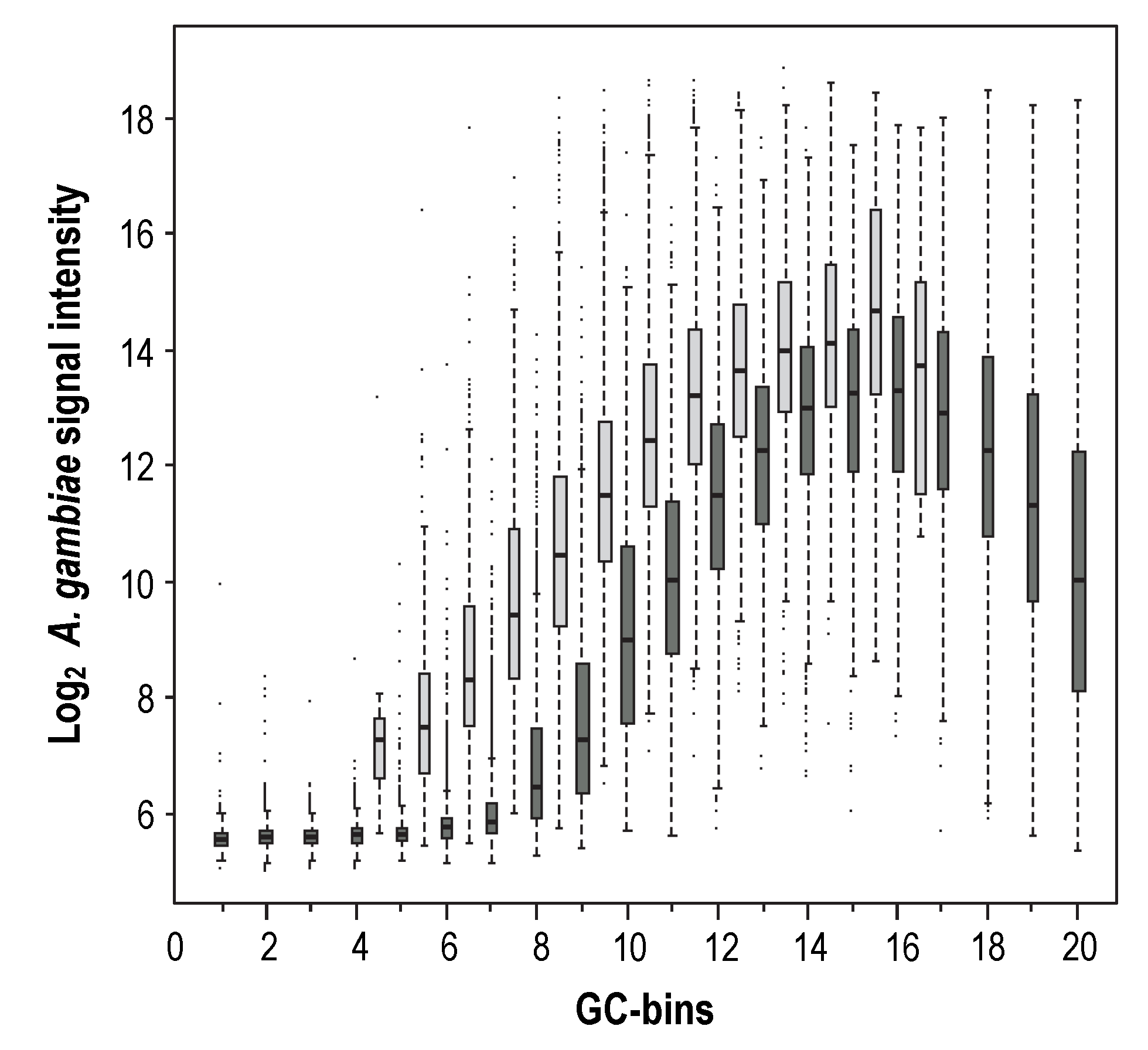

Supplement: Figure S1 — aCGH signal intensities of the control probes according to GC-content. Box- and-whisker plot showing the log2 A. gambiae signal intensity distributions of the positive control probes (light grey) and the negative control probes (dark grey) for 20 GC-bins, each bin corresponding to a GC-content increase of 5%. (TIFF) [file pone.0048096.s001.tiff]

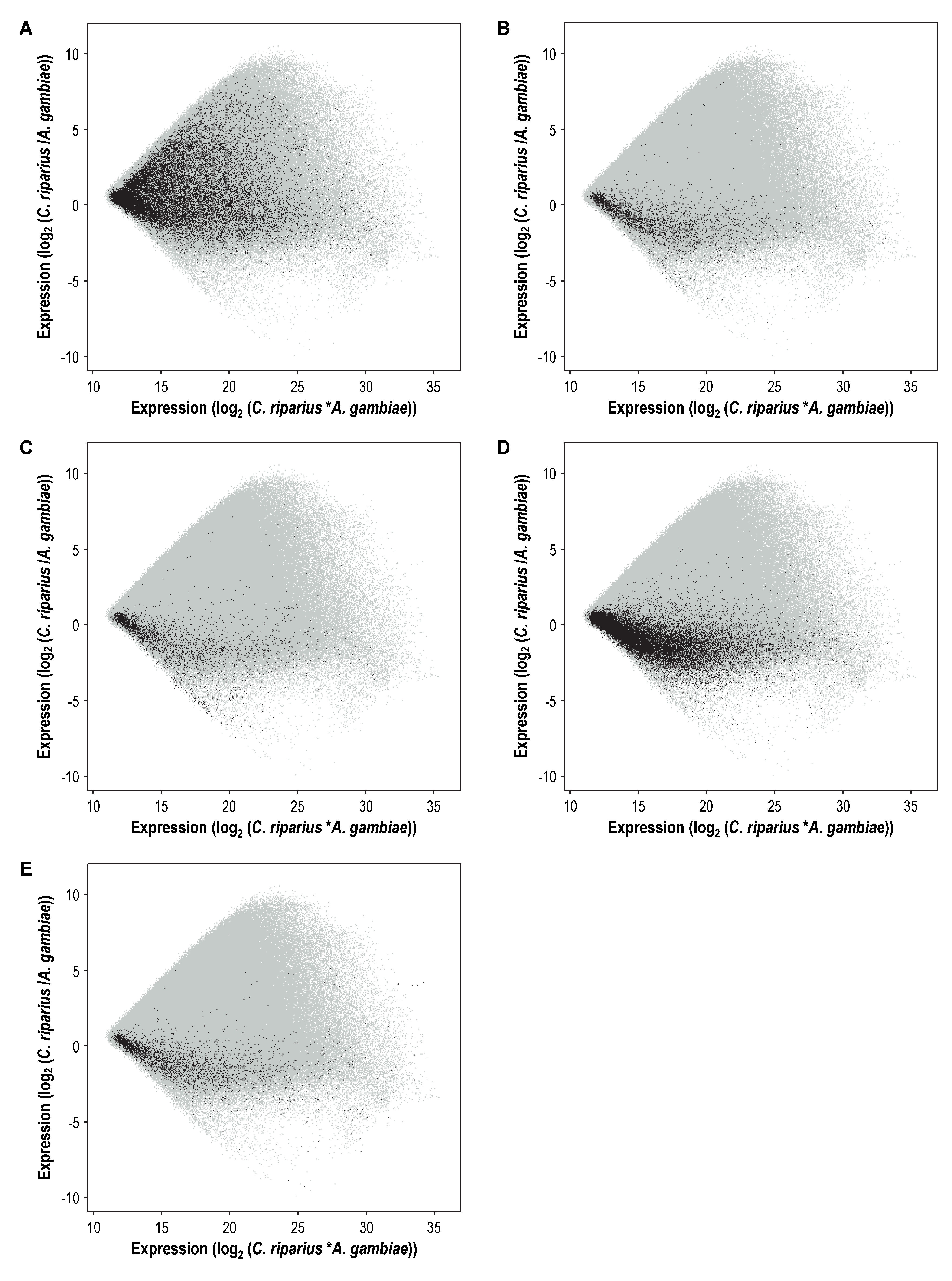

Supplement: Figure S2 — MA-plots of the aCGH experiment obtained for the various dipteran probe collections. The grey dots represent the entire probe library, except the negative control probes. The black are the probes targeting the ESTs of respectively (A) Chironomus spp., (B) Anopeheles darlingi, (C) Anopheles funestus, (D), Aedes aegypti and (E) Culex quinquefasciatus. (TIFF) [file pone.0048096.s002.tiff]
