# Supplementary material for: Combining Next-Generation Sequencing and Microarray Technology into a Transcriptomics Approach for the Non-Model Organism Chironomus riparius
Source: PLoS One. 2012 Oct 25;7(10):e48096. doi: 10.1371/journal.pone.0048096 (PMC3485019; doi:10.1371/journal.pone.0048096)
Supplement: Table S1 — Detailed C. riparius sample list. (DOCX) [file pone.0048096.s003.docx]

**Table S1: Detailed *C. riparius* sample list.**

| **Specimens** | **Pre-exposed**  **(3 generations)** | **Exposed**  **(14 days)** | **Time/ Dose range** | **Number** |
| --- | --- | --- | --- | --- |
| ***Developmental stages*** | |  |  |  |
| Egg ropes | n.a | n.a | <1h post laying | 4 |
| Egg ropes | n.a | n.a | 24h post laying | 4 |
| Egg ropes | n.a | n.a | 48h post laying | 4 |
| Egg ropes | n.a | n.a | 72h post laying | 4 |
|  |  |  |  |  |
| 1^st^ instar larvae | n.a | n.a | <1 day post hatching | 4 |
| 2^nd^ instar larvae | n.a | n.a | 4 day post hatching | 4 |
| 3^rd^ instar larvae | n.a | n.a | 8 day post hatching | 4 |
| 4^th^ instar larvae | n.a | n.a | 14 days post hatching | 4 |
|  |  |  |  |  |
| Pupae | n.a | n.a | 14-16 days post hatching | 4 |
|  |  |  |  |  |
| Adult males | n.a | n.a | <1h post emerging | 4 |
| Adult males | n.a | n.a | 24h post emerging | 4 |
| Adult males | n.a | n.a | 48h post emerging | 4 |
| Adult males | n.a | n.a | 60h post emerging | 4 |
|  |  |  |  |  |
| Adults females | n.a | n.a | <1h post emerging | 4 |
| Adults females | n.a | n.a | 24h post emerging | 4 |
| Adults females | n.a | n.a | 48h post emerging | 4 |
| Adults females | n.a | n.a | 60h post emerging | 4 |
| ***Toxicant exposed larvae*** | |  |  |  |
| Larvae | n.a | Cadmium | 0.5, 1.0, 2.0, 4.0 mg Cd / kg dw | 4 |
| Larvae | n.a | Copper | 10, 20, 30, 40 mg Cu/ kg dw | 4 |
| Larvae | n.a | Tributyltin | 0.5, 1.0, 2.0, 4.0 mg Sn / kg dw | 4 |
| Larvae | n.a | Phenanthrene | 50, 100, 200, 400 mg Phe/ kg dw | 4 |
|  |  |  |  |  |
| Larvae | Cadmium | Cadmium | 0.5, 1.0, 2.0, 4.0 mg Cd / kg dw | 4 |
| Larvae | Copper | Copper | 10, 20, 30, 40 mg Cu/ kg dw | 4 |
| Larvae | Tributyltin | Tributyltin | 0.5, 1.0, 2.0, 4.0 mg Sn / kg dw | 4 |
| Larvae | Phenanthrene | Phenanthrene | 50, 100, 200, 400 mg Phe/ kg dw | 4 |

* n.a.: not applicable
