# Supplementary material for: Combining Next-Generation Sequencing and Microarray Technology into a Transcriptomics Approach for the Non-Model Organism Chironomus riparius
Source: PLoS One. 2012 Oct 25;7(10):e48096. doi: 10.1371/journal.pone.0048096 (PMC3485019; doi:10.1371/journal.pone.0048096)
Supplement: Table S2 — blastx results using different e-value cut-offs. (DOCX) [file pone.0048096.s004.docx]

**Table S2: Number of transcripts (percentage of total) with blastx match using different e-value cut-offs.**

| **Threshold** | **# Isotigs** | **# Singletons** |
| --- | --- | --- |
| 1e-3 | 16,824 (71.0%) | 24,129 (17.9%) |
| 1e-6 | 16,036 (67.6%) | 19,986 (14.8%) |
| 1e-10 | 15,192 (64.1%) | 15,463 (11.4%) |
| 1e-30 | 11,883 (50.1%) | 4,235 (3.1%) |
| 1e-50 | 9,244 (38.9%) | 794 (0.6%) |
