# Supplementary material for: Combining Next-Generation Sequencing and Microarray Technology into a Transcriptomics Approach for the Non-Model Organism Chironomus riparius
Source: PLoS One. 2012 Oct 25;7(10):e48096. doi: 10.1371/journal.pone.0048096 (PMC3485019; doi:10.1371/journal.pone.0048096)
Supplement: Appendix S1 — Additional information to the Materials and Methods. (DOCX) [file pone.0048096.s006.docx]

**Appendix S1: Supplementary Materials and Methods**

## cDNA library construction and next-generation sequencing

An aliquot of the total RNA sample was send to GATC Biotech (Konstanz, Germany) where a normalized cDNA library was prepared and sequenced using Titanium chemistry on a GS FLX Instrument (Roche Diagnostics) according to manufacturer’s protocol. PolyA^+^ RNA was isolated with the Dynabeads^®^ mRNA Purification kit (Invitrogen), fragmented with an ultrasound pulse of 30 seconds at 4˚C and used to synthesize randomly primed first-strand cDNA. The 454 adapters A and B were subsequently ligated to the 5' and 3' ends of the cDNA, using additional GATC Biotech adapters. After amplification with 19 PCR cycles of the adaptor-ligated-cDNA, normalization was carried out by one cycle of denaturation and re-association of the cDNA. After discarding the re-associated double-stranded cDNA, the normalized single-stranded cDNA was purified with hydroxyapatite chromatography and amplified with 10 PCR cycles. Normalized cDNA in the size range of 450 to 700 bp was selected using gel fractionating and used for pyro-sequencing (two times half a PicoTiter plate). All critical steps during the cDNA library preparation were analysed with the MCE^®^-202 MultiNA Microchip Electrophoresis System (Shimadzu Biotech).

## Functional annotation of the transcriptome

The *C. riparius* isotigs and singletons were functionally annotated using the Blast2GO^®^ suite (Götz et al., 2008). The transcripts were searched against the GenBank non-redundant database at the National Center for Biotechnology Information (NCBI) (release of March 14 2011) using blastx (Altschul et al., 1997). Alignments with e-value < 1e^-3^ were considered significant and up to 10 blastx hits per transcript were taken into account. Transcripts were assigned protein accession numbers based on their best blastx hit. Annotation was done by retrieving the Gene Ontology (GO) terms (Ashburner et al., 2000) associated with the 10 blastx hits per transcript, and applying the Blast2GO annotation rule using the default settings (Conesa et al., 2005). After an InterProScan protein domain search (Zdobnov and Apweiler, 2001) and the application of the annotation augmentation tool ANNEX (Myhre et al., 2006) the final annotation was obtained. We reduced the complexity of the obtained GO terms by applying the general GO-slim terminology, which consists of a subset of the GO vocabulary encompassing key ontological terms. Annotated transcripts were furthermore assigned enzyme commission (EC) numbers, so the Kyoto Encyclopedia of Genes and Genomes (KEGG) database (Ogata et al.,1999) could be searched for associated pathways.

## Array-based comparative genomic hybridization (aCGH)

***gDNA extraction.*** Extraction of gDNA from 30 pooled fertilized *C. riparius* egg ropes, respectively, 30 unfed *A. gambiae* adults, was conducted according to (Brunner et al., 2010). The two samples were pulverized in liquid nitrogen, divided over 2 mL microcentrifuge tubes (300 µL powder/ tube) and mixed for 30 min at 65°C with 900 µL of 65°C CTAB extraction buffer made to 2% (v/v) 2-mercaptoethanol before use (100 mM Tris-Cl (pH 8.0), 2.0 M NaCl, 20 mM EDTA (pH 8.0), 3% (w/v) CTAB (H6269, Sigma-Aldrich), 2% (w/v) PVP-40 (PVP40, Sigma-Aldrich). The homogenates were extracted for 30 min with an equal volume of chloroform/isoamylalcohol (96:4), after which they were centrifuged for 15 min at 5.000 g. The aqueous phases were recovered and DNA was precipitated with an equal volume of ice-cold isopropanol. After 30 min of incubation at -20°C, DNA was pelleted at 12.000 RPM for 15min. DNA pellets were washed with ice-cold 70% ethanol, centrifuged at 12.000 RPM for 5 min, air dried and resuspended in 100 μL TE buffer (10 mM Tris-Cl (pH 8.0)). To increase efficiency in the subsequent steps, all DNA samples belonging to the same species were pooled together and the end volume was adjusted to 1 mL with TE buffer. Residual RNA was removed by adding 3 μL RNAse A (10 mg/ml, R6513, Sigma-Aldrich) and overnight incubation at 37°C. DNA was re-extracted with an equal volume of phenol/chloroform/isoamylalcohol (25:24:1), followed by centrifugation at 8.000 RPM for 15 min. The recovered aqueous phase was treated with an equal volume of chloroform/isoamylalcohol (96:4) and centrifuged at 12.000 RPM for 10 min. The final aqueous phase was treated with an equal volume of 100% ethanol and 1/10 volume of 3 M sodium acetate (pH 5.2) and incubated for 30 min at -20°C. DNA was pelleted for 15 min at 12.000 RPM, air dried after decanting of residual liquid and washed with ice-cold 70% ethanol. DNA pellet was spun down at 12.000 RPM for 5 min and, after air drying, resuspended in 100 μL TE buffer. DNA quality and quantity were determined with gel electrophoresis (0.5% agarose in TAE buffer) and NanoDrop ND-1000 UV-VIS spectrophotometer measurements.

***gDNA amplification and labelling.*** gDNA was amplified and labelled by strand displacement amplification. 200 ng DNA was combined with 5 µg random octamers (Biolegio). The DNA was partly fragmented by heat at 99°C for 10 min and allowed to cool on ice for 5 min. The DNA/octamer mix was made on ice to 50 mM Tris-Cl (pH 7.5), 5 mM MgCl2, 1 mM DTT, 1 mM dGAC (GE Healthcare), 0.6 mM dTTP (GE Healthcare), 0.4 mM amino-allyl dUTP (TriLink Biotechnologies) and finally 10U Klenow exo- (Jena Bioscience) was added. After gentle vortexing the samples were place at 37°C for 8h and subsequently incubated for 15 min at 75°C to inactivate the enzyme. Each reaction was made to 1.5M sodium acetate (pH 5.2) and the amplified DNA was purified with the QIAquick PCR Purification Kit (Qiagen) according to the manufacturer’s instructions with the exception that 7 volumes of buffer PB were used instead of 5 to increase the recovery of smaller fragments. Concentrations of amplified products were measured on the NanoDrop ND-1000 and qualified on the BioAnalyzer with the DNA 1000 Kit (Agilent Technologies). From each sample 5 µg aliquots were taken, dried down and dissolved in 50 mM carbonate buffer (pH 8.5). Individual vials of Cy3/Cy5 from the mono-reactive dye packs (GE Healthcare) were dissolved in 200 µL DMSO. To each sample, 10 µL of the appropriate CyDye dissolved in DMSO was added and the mixture was incubated for 1h. Reactions were quenched with the addition of 5 µL 4M hydroxylamine (Sigma-Aldrich). This mixture was made to 0.75M Ammonium acetate and 0.2 µg/µL glycogen (Ambion). After mixing 2.5 vol. of 100% EtOH was added and mixed. All samples were incubated for at least 60 min at -20°C to enhance precipitation and centrifuged for 30 min at 13.000 g. The supernatants were carefully removed by pipetting and 1 vol. of 80% EtOH was added to the pellets. After brief vortexing the labelled DNA was pelleted once more by centrifugation for 10 min at 13.000 g. The supernatants were removed by pipetting and the pellets were dried by vacuum concentrating. Final labelled pellets were dissolved in 30 µL H_2_O and the yield and CyDye incorporation were measured with the NanoDrop ND-1000.

***Microarray hybridization and processing.*** The 1M probe-selection microarray was hybridized with 10 µg of Cy3 labelled *C. riparius* DNA and 10 µg Cy5 labelled *A. gambiae* DNA according to the Oligonucleotide Array-Based CGH for Genomic DNA Analysis manual (Agilent Technologies version 6.3). Briefly, labelled DNA was combined with 52 μL 10 × Blocking Agent and 260 μL 2× Hi-RPM Hybridization Buffer (Oligo aCGH Hybridization Kit, Agilent Technologies) in a total volume of 520 μL. The hybridization sample was incubated at 95°C for 3 min, spun down and hybridized at 37°C for 30 min. The samples was spun down and 490 μL was loaded onto a 1x1M backing in a SureHyb hybridization chamber (Agilent Technologies) and a 1M probe-selection validation microarray was placed on top. Hybridization was performed at 65°C for 40 h and 20 RPM in a hybridization oven (G2545A, Agilent Technologies). After hybridization the backing was removed and the microarray was washed with Oligo aCGH Wash Buffer 1 and 2 (Oligo aCGH Wash Buffer Kit, Agilent Technologies) according to manufacturer’s recommendations. The microarray was scanned in an ozone-free room on an Agilent G2505CA scanner at 3 μm resolution. The data was extracted with Feature Extraction version 10.7.3.1 (Protocol CGH_107_Sep09). The log_2_ transformed median signals were analysed in R (www.r-project.org).

## Array-based gene-expression (aGE)

***mRNA amplification and labelling.*** 200 ng RNA of the pooled RNA sample, of which an aliquot was pyrosequenced, was taken as input for both a regular, as well as, a modified linear RNA amplification. The regular RNA amplification was conducted with the Agilent Low RNA Input Linear Amplification Kit (Agilent Technologies) according to manufacturer’s recommendations. The modified reaction was conducted using the same kit, however, the first step where the mRNA is primed with an oligo (d)T-T7 primer and converted into double-stranded cDNA with the M-MLV reverse transcriptase, was modified. Instead of the provided 10 mM dNTP mix (10 mM dATP, 10 mM dCTP, 10 mM dGTP and 10 mM dTTP), a custom nucleotide mix was used that contained dNTP’s and dideoxynucleotides (ddNTP’s) in the ratio 4:1 (8 mM dATP, 8 mM dCTP, 8 mM dGTP, 8 mM dTTP, 2mM ddATP, 2mM ddCTP, 2mM ddGTP and 2mM ddTTP). This modification was expected to yield truncated ds cDNA since incorporation of a ddNTP will immediately terminate cDNA elongation. From here on the RNA amplification, i.e. the in vitro transcription (IVT) reaction where amplified RNA is synthesized with the T7 RNA polymerase, was conducted according to the Agilent Low RNA Input Linear Amplification Kit. Amplified RNA was purified with the E.Z.N.A.^®^ MicroElute RNA Clean-up Kit (Omega Bio-Tek), eluted with nuclease-free water and checked for quality and quantity using Bioanalyzer and NanoDrop measurements. For the CyDye labelling, 5 μg of the amplified RNA was dried in the speedvac and thoroughly redissolved in 5 μL 50 mM carbonate buffer (pH 8.5). Individual vials of Cy3/Cy5 from the mono-Reactive Dye Pack (GE Healthcare) were dissolved in 200 µL DMSO. To each sample, 10 µL of the appropriate CyDye dissolved in DMSO was added and the mixture was incubated for 1 h at room temperature in the dark. The reactions were quenched by the addition of 5 µL 4 M hydroxylamine (Sigma-Aldrich) for 15 min in the dark. The labelled RNA was purified with the E.Z.N.A. MicroElute RNA Clean Up Kit and eluted twice in 16 μL nuclease-free water. The yield and dye incorporation were measured with the NanoDrop ND-1000 in the Microarray Measurement Mode.

***Microarray hybridization and processing.*** The 1M microarray was hybridized with 2.5 µg Cy3 labelled regularly amplified RNA and 2.5 µg Cy5 labelled alternatively amplified RNA according to the Two-Color Microarray-Based Gene-Expression Analysis manual (Agilent Technologies version 6.5). Briefly, labelled RNA was fragmented by addition of 50 μL 10x Blocking agent and 10 μL 25x Fragmentation Buffer (Gene-Expression Hybridization Kit, Agilent technologies) followed by incubation at 60°C for 30 min. The sample was immediately placed on ice and, after 1 min, 250 µL 2x GE Hybridization Buffer HI-RPM was added. After the sample was mixed carefully and spun down, 490 µL was loaded onto a 1x1M backing in a SureHyb hybridization chamber and a *C. riparius* transcriptome validation microarray was placed on top. The RNA was allowed to hybridize for 17 h at 65°C and 10 RPM in a hybridization oven (G2545A, Agilent Technologies). After removal of the backing, the microarray was washed using the Gene-Expression Wash Buffer Kit (Agilent Technologies) and scanned in an ozone-free room on an Agilent G2505CA scanner at 3 μm resolution. The data was extracted with Feature Extraction version 10.7.3.1 (Protocol GE2_107_Sep09). The log_2_ transformed median signals were analysed in R.

## References

1. Altschul SF, Madden TL, Schaffer AA, Zhang J, Zhang Z, et al. (1997) Gapped BLAST and PSI-BLAST: a new generation of protein database search programs. Nucleic Acids Res 25: 3389-3402.
2. Ashburner M, Ball CA, Blake JA, Botstein D, Butler H, et al. (2000) Gene ontology: tool for the unification of biology. The Gene Ontology Consortium. Nat Genet 25: 25-29.
3. Götz S, Garcia-Gómez JM, Terol J, Williams TD, Nagaraj SH, et al. (2008) High-throughput functional annotation and data mining with the Blast2GO suite. Nucleic Acids Res 36: 3420-3435.
4. Conesa A, Götz S, Garcia-Gómez JM, Terol J, Talon M, Robles M (2005) Blast2GO: a universal tool for annotation, visualization and analysis in functional genomics research. Bioinformatics 21: 3674-3676.
5. Myhre S, Tveit H, Mollestad T, Laegreid A (2006) Additional gene ontology structure for improved biological reasoning. Bioinformatics 22: 2020-2027.
6. Ogata H, Goto S, Sato K, Fujibuchi W, Bono H, Kanehisa M. (1999) KEGG: Kyoto Encyclopedia of Genes and Genomes. Nucleic Acids Res. 27: 29-34.
7. Zdobnov EM, Apweiler R (2001) InterProScan - an integration platform for the signature-recognition methods in InterPro. Bioinformatics 17: 847-848
8. Brunner J, Wittink FRA, Jonker MJ, de Jong M, Breit TM, et al. (2010) The core genome of the anaerobic oral pathogenic bacterium *Porphyromonas gingivalis*. BMC Microbiol 10:252.
